# *In-vitro* and *in-silico* analyses of the thrombolytic potential of green kiwifruit

**DOI:** 10.1038/s41598-024-64160-y

**Published:** 2024-06-14

**Authors:** Reinhard Pinontoan, Jonathan Suciono Purnomo, Elvina Bella Avissa, Jessica Pricilla Tanojo, Moses Djuan, Valerie Vidian, Ariela Samantha, Juandy Jo, Eden Steven

**Affiliations:** 1 Department of Biology, Universitas Pelita Harapan, Tangerang, 15811 Indonesia; 2 Center of Excellence Applied Science Academy, Sekolah Pelita Harapan Lippo Village, Tangerang, 15810 Indonesia; 3 Mochtar Riady Institute for Nanotechnology, Lippo Karawaci, Tangerang, 15810 Indonesia; 4 Emmerich Research Center, Jakarta, 14450 Indonesia

**Keywords:** Actinidin, Fibrinolytic activity, Green kiwifruit, Molecular docking, Molecular dynamics, Thrombolysis, Proteases, Protein structure predictions

## Abstract

Cardiovascular diseases (CVDs), mainly caused by thrombosis complications, are the leading cause of mortality worldwide, making the development of alternative treatments highly desirable. In this study, the thrombolytic potential of green kiwifruit (*Actinidia deliciosa* cultivar Hayward) was assessed using *in-vitro* and *in-silico* approaches. The crude green kiwifruit extract demonstrated the ability to reduce blood clots significantly by 73.0 ± 1.12% (*P* < 0.01) within 6 h, with rapid degradation of Aα and Bβ fibrin chains followed by the *γ* chain in fibrinolytic assays. Molecular docking revealed six favorable conformations for the kiwifruit enzyme actinidin (ADHact) and fibrin chains, supported by spontaneous binding energies and distances. Moreover, molecular dynamics simulation confirmed the binding stability of the complexes of these conformations, as indicated by the stable binding affinity, high number of hydrogen bonds, and consistent distances between the catalytic residue Cys25 of ADHact and the peptide bond. The better overall binding affinity of ADHact to fibrin chains Aα and Bβ may contribute to their faster degradation, supporting the fibrinolytic results. In conclusion, this study demonstrated the thrombolytic potential of the green kiwifruit-derived enzyme and highlighted its potential role as a natural plant-based prophylactic and therapeutic agent for CVDs.

## Introduction

Cardiovascular diseases (CVDs) are responsible for the highest number of deaths worldwide. The World Health Organization (WHO) reported a significant increase in global deaths attributed to CVDs from 2000 to 2019, with mortality rates reaching 32% of all deaths worldwide in 2019^1,2^. CVDs are primarily caused by thrombi that are not correctly dissolved by the fibrinolytic system in the human body^3^. The formation of thrombi, although essential to the wound healing process, also entails several CVDs complications under imbalanced hemostasis, including myocardial infarction, stroke and venous thromboembolism^4^. Therefore, it is necessary to suppress and/or eliminate thrombus formation using thrombolytic or fibrinolytic agents to restore hemostasis.

Numerous medications have been employed for the purpose of preventing and treating thrombus formation, either by suppressing or eliminating its occurrence. Some examples of commonly used preventative measures include administration of platelet aggregation inhibitors such as aspirin and P2Y_12_, receptor inhibitors (G protein-coupled receptors responsible for platelet aggregation^5^), as well as anticoagulants such as warfarin^6^. In contrast, therapeutic treatments typically involve the use of thrombolytic agents such as Tissue Plasminogen Activator (tPA), streptokinase, alteplase, and tenecteplase, which are designed to dissolve existing thrombus formations^7,8^. However, the current commercial fibrinolytic agents have some limitations, including low fibrin specificity, allergic reactions, and a relatively high cost^9^.

Various studies have attempted to identify alternative substances with thrombolytic or fibrinolytic activities in natural sources. Exploring the potential benefits of these natural sources not only expands our knowledge of cardiovascular health benefits associated with consuming such products but also offers the potential to discover new drugs with improved thrombolytic efficacy and reduced side effects. Natto, a conventional Japanese cuisine derived from the fermentation of soybeans by *Bacillus subtilis,* is a traditional cheese-like product widely consumed in Japan and has been linked to a decreased risk of cardiovascular diseases^10^. Its health benefits are mainly attributed to nattokinase, an enzyme secreted by *B. subtilis*, which has been demonstrated to possess fibrinolytic activity, in addition to other beneficial effects, such as anti-coagulation, anti-atherosclerosis, and antihypertension^11,12^. Most notably, the antithrombotic activity of nattokinase is widely recognized and does not result in any significant adverse effects when consumed. Some studies have suggested that nattokinase may surpass aspirin^13^. Comparable to fermented foods, plant-based diets have also emerged as a potential source of thrombolytic agents. Although fruit and vegetable consumption has gained some interest for their health benefits related to CVDs, there is still limited information regarding the fibrinolytic potential of fruits^14^. One fruit that has shown a particular benefit to human metabolic health for CVDs markers is kiwifruit^15^.

Green kiwifruit (*Actinidia deliciosa* cultivar Hayward) contains many compounds that provide health benefits, including nutritional, digestive, and overall metabolic health. Some studies have suggested that green kiwifruit confers health benefits on irritable bowel syndrome, diabetes, and CVDs^15^. Its rich components include antioxidants, vitamin C, minerals, and large amounts of highly active proteolytic enzymes^16^, including actinidin. Notably, actinidin is a cysteine protease similar to the proteases found in other fruits, such as pineapples, figs, and papayas^17^. The proteolytic mechanism of actinidin has been suggested to be similar to that of papain^18^.

Green kiwifruit has been reported to have fibrinolytic activity^19,20^. However, no study has investigated the thrombolytic activity of green kiwifruit against human fibrin at the molecular level. To the best of our knowledge, there have also been no studies on the molecular mechanisms underlying the proteolytic activity of actinidin. Therefore, this study aimed to examine the thrombolytic potential of an actinidin-containing green kiwifruit extract *in-vitro* using human fibrin as its substrate and to predict the fibrinolytic mechanism of action of actinidin at the molecular level using *in-silico* approaches.

## Results and discussion

In this study, the thrombolytic potential of green kiwifruit was assessed through both *in-vitro* and *in-silico* analyses. To accomplish this, whole blood clot lysis and human fibrin degradation assays were employed to assess the thrombolytic and fibrinolytic activities of kiwifruit enzymes. Additionally, molecular docking and molecular dynamics analyses were applied to predict the fibrinolytic mechanism through molecular interactions between the enzyme and fibrin peptides.

### Phytochemical analyses of green kiwifruit extract

Green kiwifruit contains a primary protease identified as actinidin, which is believed to be responsible for conferring cardiovascular health benefits^15,19,20^. To separate this enzyme from other components of green kiwifruit, an acetone-based protein precipitation method was employed. The resulting extract was used for the *in-vitro* assays employed in this study. In addition to isolating proteins through acetone precipitation, it is crucial to analyze other phytochemicals that may co-precipitate with them. We thus analyzed the acetone-precipitated green kiwifruit extract using LC-MS/MS-QTOF.

We targeted six groups of natural product categories commonly found in green kiwifruit: alkaloids, flavonoids, organic acids/esters, phenols, polyphenols, and terpenoids. The results, detailed in Table S1, revealed the presence of alkaloids, organic acids (esters), and terpenoid groups. Further analysis identified seven alkaloids [(3-Methoxycarbonylamino-2-methylphenyl)-cabamic acid methyl ester, 19-epi-3-lso-ajmalicinem Adenine, Denudatine, Gentiatibetine, Guanine, and Isopteropodic acid], one ester [(Z,Z,Z)-9,12,15-octadecatrienoic acid methyl ester] and five terpenoids (E-p-Coumatic acid, Esculentoside A, Ginsenoside F1, Nigakilactone H, and Picrasinoside G) that were co-precipitated alongside the proteins of green kiwifruit. However, to date, no reports have confirmed the correlation between the detected phytochemicals and thrombolytic activity. This indicates that other phytochemicals in the extract may not interfere with the thrombolytic activity of the crude enzyme extract of green kiwifruit.

### Whole blood clot lysis test

A whole blood clot lysis test was performed to assess the ability of protease extracted from green kiwifruit to lyse whole blood clots. Crude enzyme extract samples were prepared at concentrations of 100, 75, and 50%, with protein concentrations of 6.0, 4.5, and 3.0 mg/mL, respectively.

**Figure 1:**
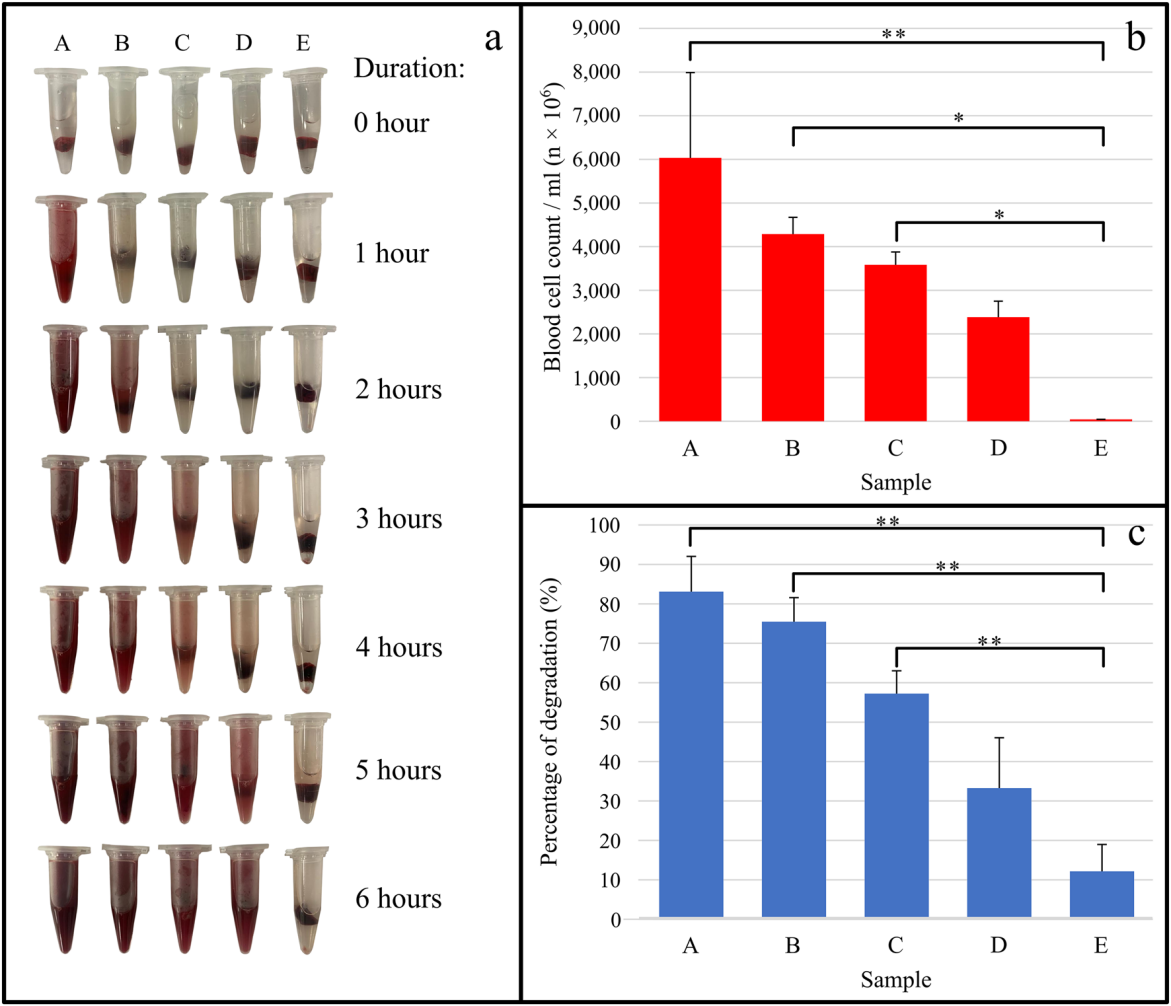
Whole blood clot lysis test using green kiwifruit crude enzyme extract. (**a**) Different samples incubated with whole blood clots for up to 6 h at 37 ℃. (**b**) Total counts of released red blood cells after 6 h of incubation at 37 ℃. (**c**) Percentage of blood clot degradation after 6 h of incubation at 37 ℃. Whole blood clots treated with (**A**) 50 FU/mL nattokinase (positive control) and (**B**) 6.0 mg/mL, (**C**) 4.5 mg/mL, and (**D**) 3.0 mg/mL crude green kiwifruit enzyme extract. (**E**) Whole blood clot treated with phosphate-buffered saline (negative control). Vertical bars indicate standard error. Significant differences are indicated with * (*P* < 0.05) and ** (*P* < 0.01).

Figure 1 depicts the thrombolytic activity of the crude enzyme extracted from green kiwifruit during whole blood clot lysis. Upon incubation of the green kiwifruit extract in the whole blood clot lysis solution, the color of the solution changed from its original hue to a redder color over time. At concentrations of 100 and 75%, the total number of released blood cells was noticeably higher (4.3 ± 0.67 × 10^9^ and 3.6 ± 0.51 × 10^9^ cells/mL, respectively, with *P* < 0.05), and the dry weight of the whole blood clots was significantly reduced (73.0 ± 1.12 and 55.0 ± 1.00%, respectively, with *P* < 0.01) compared to the negative control (total released blood cells at 43.6 ± 2.81 × 10^6^ cells/mL and a 12.0 ± 1.39 % reduced dry weight). These results demonstrate that the measured thrombolytic activity was dose-dependent, as higher enzyme concentrations corresponded to higher thrombolytic activity (Fig. 1b,c). Whole blood clot lysis results supported the notion that the crude enzyme extract from green kiwifruit contains a thrombolytic component with an activity comparable to that of nattokinase.

Several plant components have been investigated for their possible thrombolytic properties, ranging from the entire Indian pennywort plant (*Centella asiatica* L.)^21^ to the leaves of Sugandhmantri (*Homalomena aromatica*)^22^ and the stem of the peepal tree (*Ficus religiosa* L.)^23^. These studies conclude that the identified compounds target pathways associated with thrombosis. However, the specific bioactive compounds that exert thrombolytic activity have not yet been clearly defined. Conversely, our previous study suggested that the root of ginger (*Zingiber officinale*) might promote thrombolysis through fibrinolysis, potentially via proteases^24^. Therefore, further analysis was conducted to examine the potential proteolytic activity of the green kiwifruit crude enzyme extract using a fibrin degradation assay.

### Fibrin degradation assay

**Figure 2:**
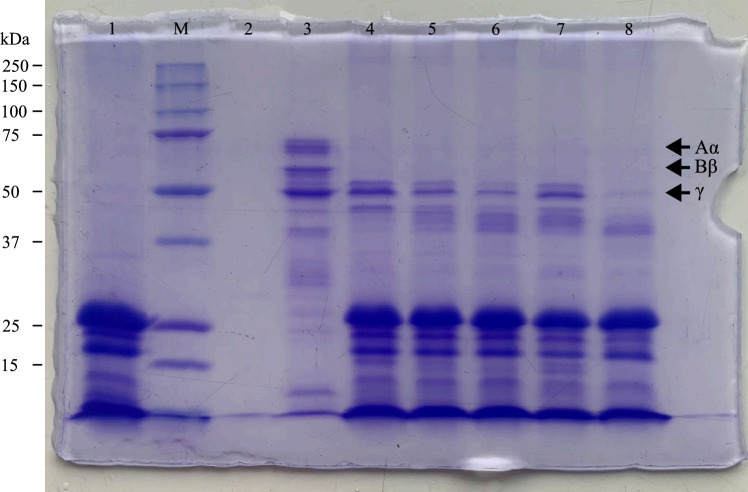
Sodium dodecyl sulfate–polyacrylamide gel electrophoresis (SDS-PAGE) analysis of fibrin degradation by the green kiwifruit extract. Lane (**M**) contained a broad range molecular weight protein marker, whereas lane (**1**) contained the crude enzyme extract from green kiwifruit, (**2**) fibrin and nattokinase incubated for 120 min, (**3**) fibrin only in phosphate-buffered saline. Lane (**4**) contained fibrin and the green kiwifruit crude enzyme extract incubated for 5 min, (**5**) 30 min, (**6**) 60 min, (**7**) 90 min, and (**8**) 120 min. Arrows indicate the Aα (70 kDa), Bβ (60 kDa), and γ (51 kDa) fibrin chains. The green box shows the gradual degradation of fibrin by the crude enzyme extract. A protein band similar in size to that of actinidin (26 kDa) was observed in lanes **1** and **4–8**.

Exploration of the fibrinolytic potential of the crude enzyme extract was performed upon confirmation of positive thrombolytic activity. The fibrin degradation assay, visualized using Sodium Dodecyl Sulfate–Polyacrylamide Gel Electrophoresis (SDS-PAGE), was employed as a more informative method than the fibrin plate assay^25^. Fibrin was produced through a 1-h incubation of fibrinogen and thrombin, followed by the addition of the crude enzyme extract from green kiwifruit to assess the rate of fibrin degradation over varying incubation durations of 5, 30, 60, 90, and 120 min. After incubation, the separated fibrin and its degraded fragments were analyzed using SDS-PAGE, as shown in Fig. 2.

Upon activation by thrombin, fibrinogen generates three non-crosslinked chains: Aα (70 kDa), Bβ (60 kDa), and γ (51 kDa; Fig. 2, lane 3)^26^. In contrast to the full fibrin chains displayed in Lane 3, the absence of both Aα and Bβ chains was observed as early as the first 5 min of enzyme-fibrin incubation (Fig. 2, lane 4), indicating the rapid degradation of Aα and Bβ chains by the green kiwifruit enzyme. Conversely, the γ-fibrin chain persisted even after 90 min of incubation. At 120 min, although the crude enzyme extract mostly degraded the γ-fibrin chain, a thin band was still visible on the gel. This degradation rate was comparable to that of nattokinase, which degraded all fibrin chains within 120 min of incubation (Fig. 2, lane 2).

The experimental results indicated that fibrin chains were selectively degraded, suggesting a potential difference in the enzyme's substrate affinity. This pattern of selective degradation was also observed with other plant-derived proteases, including lunathrombase from *Leucas indica*, clerofibrase from *Clerodendrum colebrookianum*, ficin from *Ficus carica*, and ginger proteases^24,27–29^. Although lunathrombase and clerofibrase are serine proteases, ficin is a cysteine protease, and the protease extracted from ginger was predicted to be either zinc metalloprotease, serine proteases, or aspartic proteases; the γ-fibrin chain was found to be the most resistant to degradation. This may be attributed to the globular structure of the γ-fibrin chain, which inhibits the exposure of cleavage sites to proteases, thereby limiting the rate of proteolysis on the chain^30^. Overall, the assay provided insights into how the crude enzyme extract from green kiwifruit induces thrombolysis, which is primarily achieved through proteolytic activity targeting fibrin chains, also known as fibrinolysis.

### Structural prediction of actinidin

In this study, in-silico analyses were designed to evaluate the protease activity of actinidin on fibrin obtained *in-vitro*. However, due to the unavailability of the actinidin crystal structure from *A. deliciosa* cultivar Hayward (ADHact) in the database of the three dimensional (3D) protein structure, amino acid sequence alignments were conducted with known actinidin structures from other kiwifruit species to find a reference structure for evaluating the structural prediction accuracy. To this end, amino acids of ADHact (AAA32629.1) and amino acid sequences from known actinidin crystallographic structures from a close species of kiwifruit, *A. chinensis* (PDB: 3P5V, 3P5W, 3P5X, 3P5U, 1AEC, and 2ACT), were compared using multiple sequence alignments.

**Figure 3:**
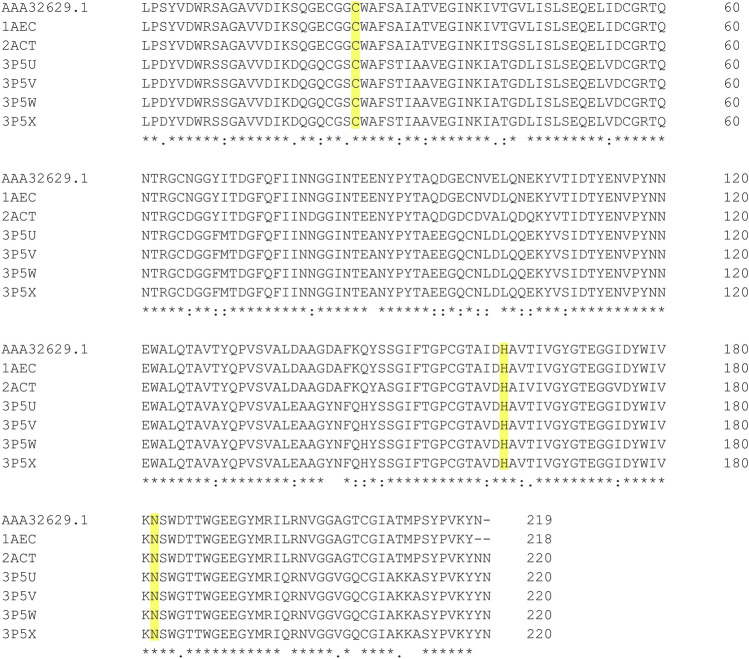
Multiple sequence alignment between amino acid residues of actinidin from *Actinidia deliciosa,* ADHact (AAA32629.1) and the known actinidin structure of *Actinidia chinensis*. All retrieved amino acid sequences of *A. chinensis* are represented with a PDB code. The catalytic triads are highlighted in yellow. Positions with fully conserved residues are denoted by asterisks (*), positions with conservation between amino acid groups of similar properties are denoted by colons (:), and positions with conservation between amino acid groups of weakly similar properties are denoted by periods (.).

**Figure 4:**
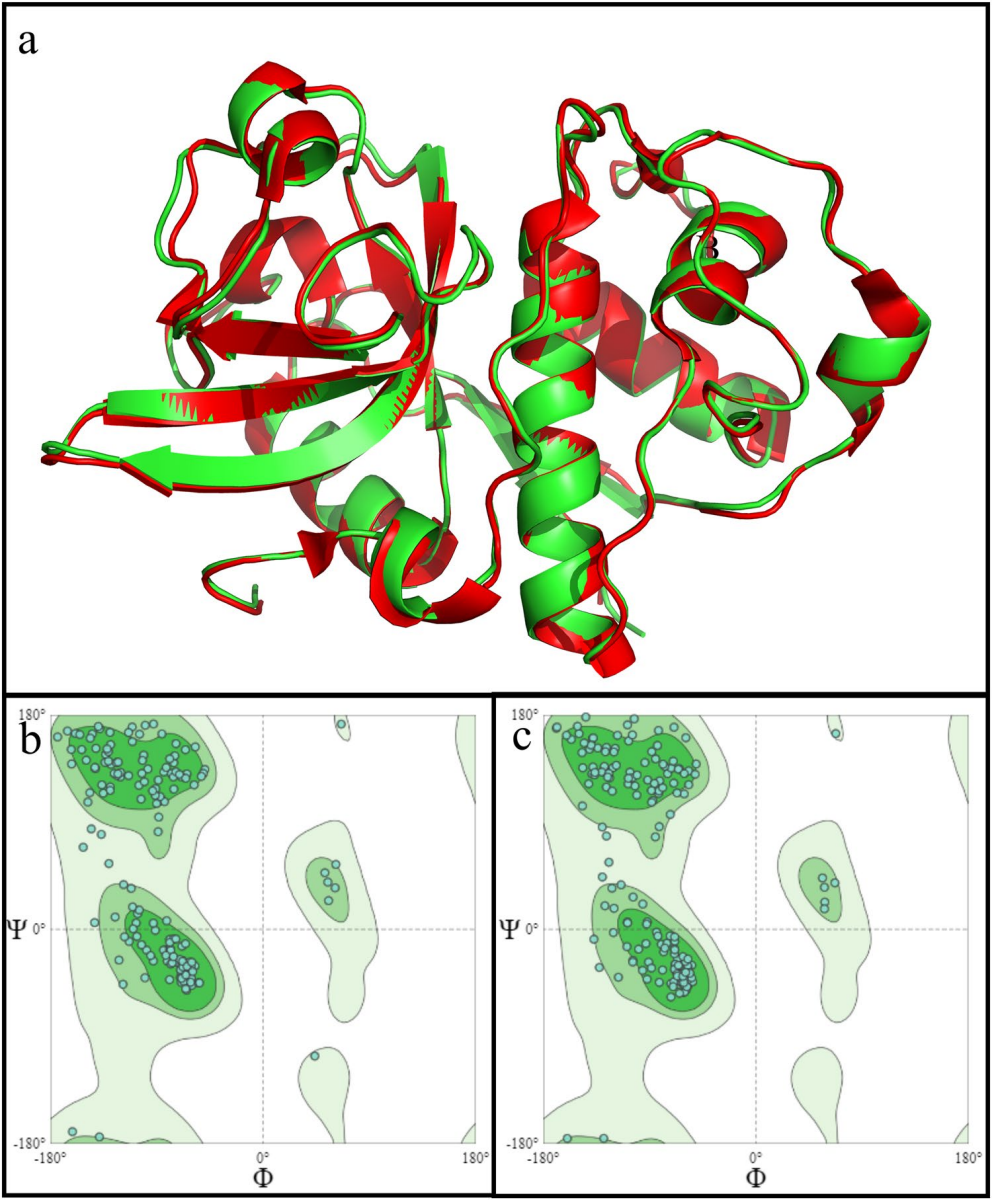
Structural comparison between predicted structure of actinidin from *Actinidia deliciosa* (ADHact) and *Actinidia chinensis* (AC_1AEC). (**a**) The structural superimposition of ADHact (green) and AC_1AEC (red) indicates high similarity between the two structures. The general Ramachandran plot of (**b**) ADHact and (**c**) AC_1AEC indicates favorable torsional angle of all amino acids in both enzymes. The three contours represent 99.7% (gray), 95.0% (light green), and 80% (dark green) of the calculated structural databases of known proteins.

Based on the multiple sequence alignment shown in Figure 3, actinidin from *A. chinensis* (PDB: 1AEC; hereafter referred to as AC_1AEC) emerged as a suitable candidate for structural evaluation because the amino acid sequence of ADHact was highly similar (99.5%) to AC_1AEC, differing by only one amino acid at position 101 (E101 in ADHact versus D101 in AC_1AEC). Next, the structure of actinidin was predicted using AlphaFold2, a protein structural prediction program based on a machine learning algorithm that was trained on known protein structures, whereafter multisequence alignments were used to predict the 3D structure of the unknown protein from its sequence^31^. The evaluation of the predicted ADHact structure was performed by superimposition calculation and Ramachandran plot measurement, compared to the known structure of the AC_1AEC, as depicted in Fig. 4.

Based on the calculated root mean square deviation (RMSD), the superimposed models from both the predicted ADHact structure and AC_1AEC protein crystal structure only resulted in a difference of 0.350 Å (Fig. 4a). Furthermore, to validate the torsional angle of the predicted structure, the Ramachandran plot of ADHact was compared with that of AC_1AEC. The resulting plot indicated that all torsional angles of ADHact (Fig. 4b) and AC_1AEC (Fig. 4c) were well within the contour range of 99.7% of known structures. The protein structural comparison of ADHact to the crystallographic structure of AC_1AEC revealed a high degree of similarity, indicating that the predicted structure is likely to have close resemblance to the natural actinidin found in *A. deliciosa*.

### Prediction of the fibrin cleavage sites

To further elucidate the molecular mechanism of fibrin degradation as well as the different rate of fibrin chain degradation, prediction of cleavage sites according to the substrate specificity of actinidin^32–34^ was performed using Random Peptide Generator (RPG) tools. The predicted ADHact structure and known human fibrin crystal structure (PDB: 2HLO) were docked using the PyRx software. Finally, interactions between the complexes were analyzed. The RPG prediction results are shown in Table 1.

**Table 1:** Fibrin cleavage sites identified using the Rapid Peptide Generator.

Fibrin chain	Cleavage sites (P1, P1’)
Aα	Arg162–Gly163 Arg171–Glu172
Bβ	Arg346–Gly347
γ	Lys406–Gln407

P1 and P1’ are the amino acids adjacent to the cleaved peptide bond at the N-terminal and C-terminal sides, respectively.

All fibrin chains were predicted to be cleavable by actinidin, in line with the fibrin degradation results shown in Figure 2. However, the sequential degradation of fibrin chains, specifically the initial cleavages at Aα and Bβ followed by those at γ, remained unaddressed. By analyzing the interaction between ADHact and fibrin peptides, a deeper understanding of the preference of the enzyme for fibrin chains might be uncovered. Therefore, we performed a molecular docking study between ADHact and fibrin peptides containing the predicted cleavage site.

### Molecular prediction of the actinidin-fibrin interaction

**Figure 5:**
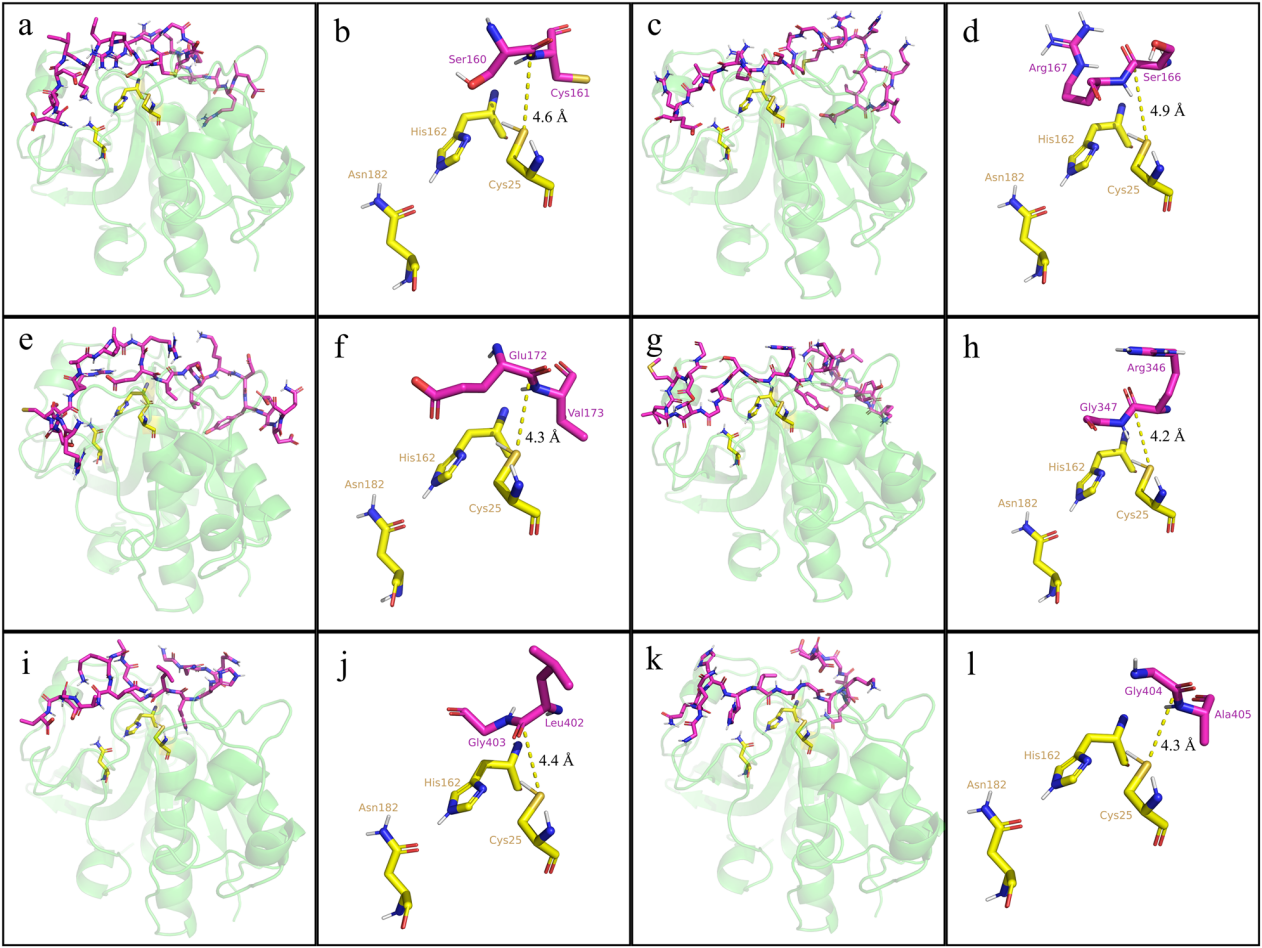
Molecular interaction between the catalytic triad of actinidin (green) and fibrin (purple). The actinidin catalytic triad (yellow), consisting of Cys25, His162, and Asn182, was predicted to cleave peptide bonds for: chain Aα at Ser160–Cys161, (**a**) whole and (**b**) simplified visualization; Ser166–Arg167, (**c**) whole and (**d**) simplified visualization; Glu172–Val173, (**e**) whole and (**f**) simplified visualization; chain Bβ at Arg346–Gly347, (**g**) whole and (**h**) simplified visualization; and chain γ at Leu402–Gly403, (**i**) whole and (**j**) simplified visualization; Gly404–Ala405, (**k**) whole and (**l**) simplified visualization. The yellow dotted line indicates the distance (Å) between the sulfur atom of Cys25 and carbon atom of the peptide bond.

Upon successful 3D modeling of the ADHact structure and the cleavage site of fibrin chains, we predicted the molecular interaction between actinidin and each chain of fibrin. Figure 5 illustrates the successful molecular interaction between the catalytic triad of ADHact (green) and fibrin peptides (purple), as indicated by the correct conformation of the ADHact Cys25 and the peptide bond^35^.

Molecular docking results revealed that the α-carbon atom of the fibrin peptide bond was located near the catalytic sites of ADHact, Cys25 and His162 (Fig. 5). These findings are consistent with the current understanding of the catalytic mechanism of plant cysteine proteases^35^. Since the amino acid, Cys25, in actinidin acted as the main nucleophilic attack site of peptide bonds, it donated its electron to the carbon of the carboxyl group. In turn, the attack destabilized the double covalent bond (C=O) between the oxygen and carbon atoms of the carboxyl group, reducing it to a single covalent bond (C–O^–^). The reactive oxygen subsequently took back an electron back from the peptide bond (C–N) and restabilized its bond with the carbon atom, consequently cleaving the peptide into two fragments. As the covalent bond between the carbon of the carboxyl group and nitrogen of the amine group was removed, His162 restabilized the amine group by creating a temporary bond. Thereafter, the fragmented peptides detached from the protease via reduction by a water molecule.

Table 2 shows the details of the molecular docking results in terms of solvent accessible surface area (SASA in Å^2^), binding affinity (ΔG in kcal/mol), dissociation constant (Kd), cleaved sites, and distances between the sulfur atom of Cys25 (SG) and the carbon atom (C) of the fibrin peptide bond (SG–C distance in Å).

**Table 2:** Molecular docking results between *Actinidia deliciosa* var. Hayward and fibrin.

Fibrin chain	SASA (Å^2^)	ΔG	Kd	Cleaved sites (P1,P1’)	Distance (Å)
Aα.1	48.1	−14.5	5.7e-11	Ser50–Cys51	4.6
Aα.2	176.2	−14.1	1.2e-10	Ser166–Arg167	4.9
Aα.3	211.7	−12.5	1.5e-09	Glu172–Val173	4.3
Bβ.1	170.8	−15.4	1.5e-11	Arg346–Gly347	4.2
γ.1	257.8	−12.3	2.1e-09	Leu402–Gly403	4.4
γ.2	114.4	−13.5	2.9e-10	Gly404–Ala405	4.3

SASA: Solvent accessible surface area of the two amino acids at the cleavage site when unbound; ΔG: Gibbs free energy binding; Kd: dissociation constant; P1 and P1’ are the amino acids adjacent to the cleaved peptide bond at the N-terminal and C-terminal sides, respectively.

**Figure 6:**
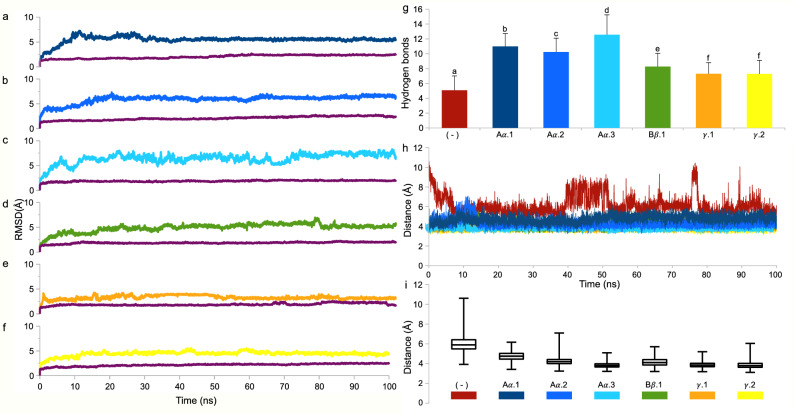
Molecular dynamic analysis of ADHact (AAA32629.1) and fibrin peptide complex. The molecular dynamic simulation was performed at 37 °C for 100 ns. The Root Mean Square Deviation (RMSD) was calculated for each the ADHact (purple) and fibrin peptide for (**a**) Aα.1 (dark blue), (**b**) Aα.2 (blue), (**c**) Aα.3 (cyan), (**d**) Bβ.1 (green), (**e**) γ.1 (orange), and (**f**) γ.2 (yellow). (**g**) Average count of hydrogen bonds between the complexes (*n* = 10,000). The negative control was obtained from the previous docking conformation that generated the largest distance between the sulfur atom of ADHact Cys25 and the carbon atom of the fibrin peptide. Different letters above the corresponding bar indicate statistical significance (*P* < 0.01) compared to the other bars. Measured distance between the sulfur atom (SG) of ADHact Cys25 and the carbon atom (C) of the fibrin peptide are represented as (**h**) distance (Å) vs. time (100 ns) and (**i**) a whisker box plot.

All cleavage sites on fibrin were accessible to ADHact, as seen from the non-zero solvent accessible surface area (SASA) described in Table 2. In addition, all interactions between ADHact and the fibrin peptides were measured to be spontaneous and occurring with a high affinity, considering the negative ΔG score and low Kd value of all docking results^36^. The measured distances between the sulfur atom of Cys25 and the carbon atom of the fibrin peptide bond varied from 4.2 to 4.9 Å (Fig. 5 and Table 2). Based on other studies, it has been suggested that 4.5 Å^37^ and 5 Å^38^ are the proposed maximum distances at which proteolytic activity can occur. Thus, the resulting docking distances support the proteolytic activity of actinidin to fibrin. However, it is noteworthy to clarify that our current results do not accurately represent the optimal distance where nucleophilic attack may occur, as proteins are dynamic in nature^39^. Thus, the conformation of ADHact might change as it binds to its ligand to facilitate stronger or weaker interactions. The low ΔG scores observed in the interaction between the fibrin chains, Aα.1, Aα.2, and Bβ.1 (Table 2), indicates a high binding spontaneity between actinidin and fibrin fragments. The high binding spontaneities between actinidin and the fibrin chains, Aα and Bβ, may explain the slower degradation pattern of chain γ^40^, as shown from the SDS-PAGE analysis results (Fig. 2). Moreover, the interaction between ADHact and fibrin chain Bβ in the docking results accurately matched the RPG-predicted cleavage site (Fig. 5G,H). The other docking results, however, were in discordance with the cleavage site predicted by the RPG (Tables 1 and 2). These results indicate the presence of possible novel catalytic sites for actinidin on fibrin. Following the static results from the molecular docking prediction, the dynamical nature of the six docked complexes was assessed using molecular dynamics simulation (Fig. 6).

The structural deviations of ADHact throughout the simulation from the six runs remained stable, with the highest RMSD score of 2.84 Å seen in the Aα.2 simulation (Fig. 6B). Ignoring the first 10 ns of the simulation, during which the complexes were still stabilizing, the cumulative deviation of the enzyme in each simulation remained below 1.4 Å. This indicates a close match between the enzyme and the reference structures^41^. Conversely, the RMSDs of all peptides were notably high, suggesting that the initial docking conformation was unstable, potentially leading to either (1) a more relaxed conformation or (2) disarrayed interactions that could cause the detachment of the peptide from the enzyme. The former may better support the molecular dynamics results, as throughout the simulation, the binding affinity of the complex stayed spontaneous around -8.8 to -15.3 kcal/mol; the average count of hydrogen bonds between ADHact and fibrin peptides stayed constant as well (Fig. 6G). Interestingly, when utilizing an incorrectly docked ADHact and peptide complex that has a large SG–C distance (7 Å) as the negative control, the total number of hydrogen bonds was significantly lower compared to the other six correct conformations (*P* < 0.01). Moreover, there was a significant difference (*P* < 0.01) between the hydrogen bonds count among different fibrin chains, with the highest observed when ADHact interacted with the Aα chain (10.2–12.6 bonds), followed by the Bβ chain (8.3 bonds) and lastly the γ chain (7.3 bonds). This suggests that the total number of hydrogen bonds between ADHact and fibrin peptides may also correlate with the faster degradation observed in the SDS-PAGE analysis (Fig. 2), as Aα and Bβ chains exhibit comparatively higher bond counts than the *γ* chain. These bonds may further stabilize the interaction and promote successful proteolytic activity^42^. Additionally, the SG–C distances during the simulation indicate high binding stability, mostly remaining within the range of 3.6 to 4.4 Å (measured from the 25th to 75th percentile). In comparison, the negative control exhibited an observable decrease in SG–C distance during the initial 10 ns of the run, but maintained a higher distance thereafter (5.5 to 6.4 Å; measured from the 25th to 75th percentile). It is plausible that ADHact attempts to correct the peptide conformation to promote proteolysis but fails due to the incompatible peptide sequence. Overall, the dynamic extrapolation of the docked results further corroborates the *in-vitro* findings, indicating the identified sequences as targets for proteolytic activity by ADHact. In hindsight, the degradation of all fibrin chains might involve other catalytic sites beyond those predicted due to limited information on the substrate specificity of actinidin. Further studies are necessary to confirm these predictions.

### Toxicity likeliness of ADHact

To serve as a potential natural remedy for preventing CVDs, ADHact must be identified as non-toxic. Although no study has reported the toxicity of actinidin, a computational analysis using Cutoff Scanning Matrix–Toxin (CSM-Toxin) tools to determine the toxicity-likeliness value of ADHact was performed, which scored ADHact with a value of 0.06. As most true non-toxin proteins typically have toxicity likeliness values below 0.05 and true positive proteins values greater than 0.95^43^, ADHact likely falls within the non-toxic category, indicating that the enzyme poses no threat.

Green kiwifruit presents a promising avenue for promoting cardiovascular health, whether through direct consumption or extraction of bioactive compounds for medicinal purposes. To provide its cardiovascular health benefits, when kiwifruit is consumed, ADHact must endure the acidic environment of the stomach and maintain its activity upon absorption into the bloodstream. Only then can ADHact work synergistically with secondary metabolites from green kiwifruit to promote cardiovascular health^19^. Indeed, a study on nattokinase pharmacokinetics demonstrated that enzyme absorption into the circulatory system is possible^44^. While the thrombolytic potency of green kiwifruit was demonstrated in this study, several questions remain to be clarified in future studies, including *in-vivo* analyses of the thrombolytic activity of the protease, and understanding the toxicity and pharmacokinetic mechanisms of green kiwifruit in the body. Hypothetically, if ADHact retains its fibrinolytic activity upon absorption into the bloodstream, it could serve as a prophylactic measure against CVDs.

## Conclusion

This study underscores the presence of the enzyme actinidin (ADHact) in green kiwifruit, which demonstrates thrombolytic effects, as evidenced by both *in-vitro* and *in-silico* analyses. Thrombolytic activity was observed in whole blood clot lysis tests, showing rapid reddening of the solution, significant liberation of blood cells (4.3 ± 0.67 × 10^9^ cells/mL) (*P* < 0.05), and a significant reduction in blood clot mass (73.0 ± 1.12%; *P* < 0.01). Furthermore, the enzyme readily degraded fibrin, with rapid digestion observed for Aα and Bβ fibrin chains within 5 min, whereas a longer duration was required for γ chain degradation (120 min). The thrombolytic activity of the green kiwifruit enzyme via fibrinolysis was further supported by *in-silico* analysis, which examined the molecular interactions between ADHact and fibrin peptides. Molecular docking revealed six favorable conformations for kiwifruit actinidin (ADHact) and fibrin chains, supported by spontaneous binding energies and distances. Moreover, molecular dynamics simulation confirmed the binding stability of the complexes of these conformations, as indicated by the stable binding affinity, high number of hydrogen bonds, and consistent distances between the catalytic residue Cys25 of ADHact and the peptide bond. The better overall binding affinity of ADHact with fibrin chains Aα and Bβ may contribute to their faster degradation, supporting the fibrinolytic results.

The results of this study have the potential to influence our understanding of the cardiovascular health benefits associated with green kiwifruit consumption and provide insights for the development of novel thrombolytic drugs. Although this study successfully elucidated the fibrinolytic potential of green kiwifruit protease, several unanswered questions require further investigation. Future studies on *in-vivo* thrombolytic activity, safety assessments, and drug administration could enhance our understanding of the benefits of green kiwifruit for human health and provide substantial evidence for its potential as a plant-based natural alternative for managing CVDs. Such findings would support the United Nations Sustainable Development Goal (SDG) number 3, which involves making good dietary choices and utilizing effective medications to ensure healthy lives and promote well-being.

## Methods

Commercial green kiwifruits (*A. deliciosa* cultivar Hayward) were purchased from a local supermarket in Tangerang, Indonesia. Fruits and plants are not categorized as endangered or at risk of extinction. All methods in this study were carried out in accordance with the relevant guidelines in the methods section.

### Preparation of green kiwifruit extracts

The green kiwifruits were blended and filtered to extract the juice, which was then separated from the pulp. The sample was centrifuged at 13,000 × g for 5 min. The supernatant was collected for partial precipitation with cold acetone at a ratio of 1:4. The sample was subsequently precipitated in acetone overnight, stored at −20 ℃, and centrifuged at 12,000 × g for 15 min. The supernatant was discarded and the pellet air-dried until the acetone evaporated. The pellet was subsequently resuspended in phosphate-buffered saline (PBS) and stored at −20 ℃ prior to testing^45^.

### LC-MS/MS-QTOF detection of green kiwifruit acetone-precipitated phytochemicals

LC-MS/MS-QTOF data acquisition and analysis were performed by PT Saraswanti Indo Genetech, using a Water Xevo-G2-S QTOF equipped with an ultra-performance liquid chromatography (UPLC) Acquity I-Class Waters. The compounds were separated using an HSS T3 pipe with a 2.1-mm diameter and 100-mm length, 1.8-µm particle size, and 100-Å pore size, using a mobile phase consisting of 0.1% formic acid in acetonitrile (A) and 0.1% formic acid in water (B) at a constant flow rate of 0.6 mL/min. A 1-μL aliquot of the extract was injected, and the gradient was set as follows: 0–16 min (1–35% B), 16–18 min (35–100% B), and 18–20 min (0% B).

Mass spectrometric analysis employed both ESI negative and positive modes with two collision energies (low 6 eV, high 15–40 eV) over a mass range of m/z 50–1200 amu. The mass spectrometer was run under the following conditions: scan time, 0.1 s; capillary voltage, 3000 V ESI+/2500 V ESI-; collision energy, low 6 eV/high 15–40 eV; source temperature, 120 °C; desolvation temperature, 500 °C; cone gas flow rate, 30 L/h; and desolvation gas flow rate, 1000 L/h. Chloramphenicol (m/z = 321.0050) and biotin (m/z = 245.0957) were used as internal standards, which resulted in −0.2 and 0.9 ppm deviations, respectively.

### Hartree–Lowry protein concentration assay

Hartree–Lowry protein concentration assay reagents were prepared to measure the protein concentration of the crude enzyme extract obtained from green kiwifruit juice. Reagent A was prepared by mixing 200 mg of sodium potassium tartrate tetrahydrate, 10 g of sodium carbonate, and 2 g of sodium hydroxide dissolved in 100 mL of distilled water. Reagent B was prepared by mixing 20 mg of sodium potassium tartrate tetrahydrate, 10 mg of copper sulfate pentahydrate, and 400 mg of sodium hydroxide dissolved in 150 mL of distilled water. Reagent C was prepared by mixing 10 mL of Folin–Ciocalteu phenol reagent in 150 mL of distilled water. A 200-μL crude enzyme sample was mixed with Reagent A and incubated in a water bath at 50 ℃ for 10 min. The sample was then mixed with Reagent B and incubated at 25 ℃ for 10 min. The mixture was subsequently mixed with Reagent C and incubated in a water bath at 50 ℃ for 10 min. A protein concentration standard curve was established by preparing a concentration series of bovine serum albumin via two-fold serial dilutions, starting at 2 mg/mL. The concentration of crude protein extract was calculated by plotting its absorbance at 650 nm against a standard curve.

### Whole blood clot lysis test

Pieces of chicken blood clots weighing 0.10–0.15 g were prepared and placed into microtubes. A crude enzyme extract (1 mL) was prepared at concentrations of 100, 75, and 50%. The samples were then transferred into microtubes containing the chicken blood clots. The blood clots were subsequently incubated at 37 °C for up to 6 h. After incubation, the dissolved clots were analyzed qualitatively by visual inspection and quantitatively by calculating the number of released blood cells per hour using a hemocytometer under a light microscope. The remaining blood clots were air-dried in an oven incubator and then weighed to obtain their final weights^46^. The percentage of blood clot degradation was calculated based on the dry weight of the clot using the following formula:

1 $$\left[\frac{initial \, weight-final \, weight}{final\, weight}\right]\times 100 \% $$

The test was replicated three times. Significance of the data (*P* < 0.05 and *P* < 0.01) was measured using one-way analysis of variance, and that between groups determined using a post-hoc Bonferroni and Holm test.

### Fibrin degradation test

One milligram of human fibrinogen (Sigma-Aldrich, St. Louis, MO, USA) was dissolved in 1 mL of PBS. Next, 10 μL of thrombin (Merck, Rahway, NJ, USA; 100 NIH U/mL) was added and the mixture incubated at 37 °C for 1 h. The PBS solution and fibrin (products of fibrinogen and thrombin) were subsequently transferred into a microtube and incubated with the sample solution at a ratio of 1:1 at 37 °C in a thermal cycler for 5, 30, 60, 90, and 120 min. Nattokinase (Doctor’s Best, Tustin, CA, USA) at a concentration of 50 FU/mL was used as the positive control. After incubation, the samples were loaded onto a polyacrylamide gel for sodium dodecyl sulfate–polyacrylamide gel electrophoresis (SDS-PAGE) analysis^45^.

### Peptide preparation

Molecular docking preparation consisted of fragmentation of the fibrin substrate using the Random Peptide Generator (RPG) as a prediction tool^47^. A novel enzyme definition was registered, as RPG did not provide the substrate specificity of actinidin in its database. The substrate specificity of actinidin was inferred from a published study on kiwellin^32^ and insulin degradation by actinidin^33,34^. Definitions of the cleavage sites are shown in Table 3. Finally, the fragmentation of human fibrin (PDB: 2HLO) was predicted.

**Table 3:** Definition of the actinidin cleavage site.

Amino acid position	Substrates			
	Kiwellin			Insulin
P4	–	–	–	G / A / S / P / E / K / R / H
P3	–	–	–	G / P / A / F / L / S / K / H
P2	–	–	–	F / A / V / L / S / M / Y / D / E
P1	T	H	R	G / A / F / Y / R / V / N / Q / E / K / H
P1’	T	S	G	G / V / L / Y / S / T / Q / D / E
P2’	–	–	–	P / F / A / V / H / G / M / T
P3’	–	–	–	G / L / F / P / V / D / E / K
P4’	–	–	–	P / A / Y / G / V / L / D / K

Amino acid positions are represented by a “P” for position and a number indicating the nth position in relation to the cleavage site. ’ indicates that the amino acid is located after the cleavage sites.

### Molecular docking

A three-dimensional (3D) protein model of human fibrin was retrieved from the Research Collaboratory for Structural Bioinformatics Database (https://www.rcsb.org/). The 3D model for actinidin used in this study was predicted using AlphaFold2 with default settings^48^. After prediction, the top sequence was relaxed using the Amber molecular dynamics software, which is included in the AlphaFold2 package. Fibrin fragments from the RPG results were concatenated into pairs with a maximum amino acid length of 20 residues (ten amino acids from both the N- and C-terminal sides). All 3D peptide structures were generated from that of human fibrin. Using AutoDock Vina^49,50^ installed in the PyRx software^51^, all ligands were docked against actinidin around a grid box centered at its catalytic triad^52^ and exhaustiveness set at 20. All docked structures were manually screened for the correct conformation between the actinidin catalytic triad and fibrin peptide bond. Visual representations of all complexes were produced using the open-source PyMol system^53^. Additionally, the binding affinities of all docked structures were predicted using Prodigy^54–56^.

### Molecular dynamics

Molecular dynamics (MD) simulations were conducted using the GROMACS version 2023.4 software^57^, employing the OPLS-AA/L force field^58^. The complexes were solvated with SPCE water molecules in a cubic box, maintaining a minimum distance of 3.0 nm between any complex atom and the box edge. Neutralization was achieved by adding sodium ions, followed by energy minimization using the steepest descent algorithm. Subsequently, the system underwent equilibration in an NVT ensemble (constant Number of particles, Volume, and Temperature) for 100 ps, employing the V-rescale thermostat to maintain a constant temperature of 298 K (25 °C). This was followed by an NPT ensemble (constant Number of particles, Pressure, and Temperature) equilibration for 300 ps, during which the system was slowly heated to 310 K (37 °C). An additional 100 ps of equilibration was performed at constant temperature and pressure. Bond lengths were constrained using the LINCS algorithm^59^, and long-range electrostatic forces were treated with the particle-mesh Ewald scheme (PME)^60^ with a grid spacing of 0.16 nm. Short-range non-bonded interactions, both Coulombic and van der Waals, were truncated at a cutoff distance of 1 nm. Finally, production MD simulations were conducted for 100 ns with a time-step of 2 fs. The RMSD, total hydrogen bonds, and distances between ADHact’s Cys25 sulfur atom (SG) and the fibrin peptide carbon atom (C) (SG–C) were calculated. The SG–C distances were further represented using a whisker box plot, showing the distribution across minimal, first quartile, median, third quartile, and maximum values.

### Toxicity likeliness

Prediction of the toxicity likeliness of ADHact was performed using the Cutoff Scanning Matrix–Toxin (CSM–Toxin) tool^43^. The amino acid sequence of ADHact was uploaded, and the resulting likeliness was obtained.

### Supplementary Information

Supplementary Information.

## Data Availability

The datasets generated during and/or analysed during the current study are available from the corresponding author on reasonable request.
