# Supplementary material for: In-vitro and in-silico analyses of the thrombolytic potential of green kiwifruit
Source: Sci Rep. 2024 Jun 14;14:13799. doi: 10.1038/s41598-024-64160-y (PMC11178772; doi:10.1038/s41598-024-64160-y)
Supplement: Supplementary file 1 — Supplementary Information. [file 41598_2024_64160_MOESM1_ESM.docx]

**Table S1.** Phytochemicals from acetone-precipitated green kiwifruit extract.

| **Category** | **Identification** | **Formula** | **RT (min)** | **Response** | **Detected MW (Da)** | **Theoretical MW (Da)** | **Mass error (ppm)** |
| --- | --- | --- | --- | --- | --- | --- | --- |
| Alkaloid | (3-Methoxycarbonylamino-2-methylphenyl)  -cabamic acid methyl ester | C_11_H_14_N_2_O_4_ | 2.57 | 2998 | 239.1032 | 238.2399 | 2.6 |
|  | 19-epi-3-lso-ajmalicine | C_21_H_24_N_2_O_3_ | 13.55 | 1081 | 353.1860 | 352.4269 | 0.2 |
|  | Adenine | C_5_H_5_N_5_ | 0.86 | 29113 | 136.0624 | 135.1267 | 4.9 |
|  | Denudatine | C_22_H_33_NO_2_ | 10.26 | 533 | 344.2571 | 343.5029 | -3.8 |
|  | Gentiatibetine | C_9_H_11_NO_2_ | 2.70 | 23999 | 166.0866 | 165.1891 | 2.0 |
|  | Guanine | C_5_H_5_N_5_O | 2.32 | 35036 | 152.0573 | 151.1261 | 4.2 |
|  | lsopteropodic acid | C_20_H_22_N_2_O_4_ | 11.88 | 923 | 355.1651 | 354.3997 | -0.4 |
| Organic Acid & Ester | (Z,Z,Z)-9,12,15-octadecatrienoic acid  methyl ester | C_19_H_32_O_2_ | 17.50 | 41563 | 293.2474 | 292.4562 | -0.3 |
| Terpenoid | E-p-Coumatic acid | C_9_H_8_O_3_ | 1.64 | 5336 | 165.0553 | 164.1580 | 3.9 |
|  | Esculentoside A | C_42_H_66_O_16_ | 10.76 | 3209 | 827.4414 | 826.9638 | -1.2 |
|  | Ginsenoside F1 | C_36_H_62_O_9_ | 12.29 | 2165 | 639.4467 | 638.8721 | 0.1 |
|  | Nigakilactone H | C_22_H_32_O_8_ | 16.88 | 28378 | 425.2147 | 424.4847 | -5.4 |
|  | Picrasinoside G | C_28_H_44_O_12_ | 5.38 | 11118 | 573.2885 | 572.6418 | -3.6 |

The ionization mode employed was Electrospray Ionization (ESI). All molecules were detected in the positive polarity as [M + H]^+^ ions. RT: retention time; MW: molecular weight (daltons).
